# On the Weierstraß form of infinite-dimensional differential algebraic equations

**DOI:** 10.1007/s00028-024-01003-3

**Published:** 2024-09-02

**Authors:** Mehmet Erbay, Birgit Jacob, Kirsten Morris

**Affiliations:** 1 IMACM, University of Wuppertal, Gaußstraße 20, 42119 Wuppertal, Germany; 2 Department of Applied Mathematics, University of Waterloo, 200 University Avenue West, Waterloo, ON N2L 3G1 Canada

## Abstract

The solvability for infinite-dimensional differential algebraic equations possessing a resolvent index and a Weierstraß form is studied. In particular, the concept of integrated semigroups is used to determine a subset on which solutions exist and are unique. This information is later used for a important class of systems, namely, port-Hamiltonian differential algebraic equations.

## Introduction

Linear differential algebraic equations (DAEs), sometimes also called *descriptor systems* or *implicit differential equations*, arise in various fields such as physics, engineering and economics. They can be written as

1 $$\begin{aligned} \frac{\hbox {d}}{{\hbox {d}}t}Ex(t)=Ax(t), \quad t\ge 0. \end{aligned}$$

Here, for complex Hilbert spaces $\mathcal {X}$ and $\mathcal {Z}$, $E:\mathcal {X}\rightarrow \mathcal {Z}$ is a bounded operator (denoted by $E\in L(\mathcal {X},\mathcal {Z})$) and $A:\mathcal {D}(A)\subseteq \mathcal {X} \rightarrow \mathcal {Z}$ is a closed and densely defined operator. We will often write (*E*, *A*) to refer to (1).

In comparison with ordinary differential equations, DAEs include both algebraic and differential constraints. Because of this, in general *E* has a non-trivial kernel. In finite dimensions it is always possible to decouple the system into an algebraic and a differential part by transforming (1) into a so-called *Weierstraß form*

2 $$\begin{aligned} \frac{\hbox {d}}{{\hbox {d}}t}\begin{bmatrix} I &  0 \\ 0 &  N \end{bmatrix} \begin{bmatrix} x_1(t)\\ x_2(t)\end{bmatrix} = \begin{bmatrix} J &  0 \\ 0 &  I\end{bmatrix} \begin{bmatrix} x_1(t) \\ x_2(t)\end{bmatrix}, \quad t\ge 0, \end{aligned}$$

where *J* is in Jordan form and *N* is nilpotent, as long as $(\lambda E-A)^{-1} \in L(\mathcal {Z},\mathcal {X})$ for some $\lambda \in \mathbb {C}$, [11, Ch. 2.1]. In this context, the nilpotency degree of *N* is called the *nilpotency index* of (*E*, *A*) [2, 11]. For finite-dimensional spaces, this definition of index coincides with other common definitions of the index. In this context, investigation of the different index definitions can be found in [5, 22, 23] for the *resolvent index*, [7, 19] for the radiality index, [2] for a general comparison of different known indices in infinite dimensions and [11, 12] for the finite-dimensional case.

However, in infinite dimensions the existence of a Weierstraß form is not guaranteed, nor is there a general procedure for calculating it. In view of this difficulty, we will specify a condition under which such a form always exists, namely the existence of the *radiality index*. One of the main uses of the Weierstraß form is to analyse the well-posedness of the system, since the DAE is divided into an algebraic part and an ODE part, see (2). For the ODE part it is possible to use known solution methods. The solvability of infinite-dimensional DAEs has been intensively studied; see for example, [5, 7, 13, 14, 17, 18, 20–23]. In [20] the splitting of $\mathcal {X}$ into $\textrm{ker}\, E$ and $\textrm{ran}\, E^*$ and the restriction of the DAE to the factorized space $\mathcal {X} / \textrm{ker}\, E$ is studied, in [7, 19] and [21] the splitting of the space, given that the radiality index exists, and the growth of the pseudo-resolvents $(\lambda E-A)^{-1} E$ and $E(\lambda E-A)^{-1}$ is analysed, in [5] a more general observation of the pseudo-resolvent growth is provided and a useful dissipativity condition for solving the DAE is presented, in [22] and [23] with the use of Wong-sequences and the resolvent index a super-set of the solution space is determined, in [18] a sufficient condition in terms of Hille-Yosida-type resolvent estimates is offered, in [14] with the help of integrated semigroups the well-posedness is investigated, and in [13], the solvability of a class of *port-Hamiltonian DAEs* is discussed.

As in [14], we will study the well-posedness of (1) using integrated semigroups. There is a strong connection between integrated semigroups and the growth rate of the resolvent $(\lambda E-A)^{-1}$, for $\lambda \in \rho (E,A)\ne \emptyset$, where

$$\begin{aligned} \rho (E,A):=\{\lambda \in \mathbb {C} \, \vert \, (\lambda E-A)^{-1} \in L(\mathcal {X},\mathcal {Z})\}. \end{aligned}$$

A densely defined linear operator *A* generates an integrated semigroup if there exists an $n\in \mathbb {N}$, such that

3 $$\begin{aligned} \left\| (\lambda I -A)^{-1} \right\| \le C \left| \lambda \right| ^{n-1}, \quad \lambda \in \mathbb {C}_{\textrm{Re}\ge \omega }, \end{aligned}$$

for some $C, \omega >0$ [15, Thm. 4.8]. For $n=0$ this matches the condition of a sectorial operator *A*, which is used for the generation of a analytic semigroup. Notice that (3) coincides with the *complex resolvent index* for $E=I$. Based on this, we build on existing solution methods for the abstract Cauchy problem $\frac{\hbox {d}}{{\hbox {d}}t}x(t)=Ax(t)$, $x(0)=x_0$ on the subspace $\mathcal {D}(A^n) = \textrm{ran}\, ((\lambda I-A)^{-1})^n$ [10, Sect. 1.3] and extend these studies to the differential algebraic Eq. (1) with $x_0 \in \textrm{ran}\,( (\lambda E-A)^{-1} E)^n$. For further details on integrated semigroups we refer to [1, 15].

We subsequently focus on a special class of systems, namely, *port-Hamiltonian differential algebraic equations* (pH-DAEs). These systems provide a framework for modelling and analysing energy-dissipating physical systems, including electrical circuits, mechanical systems, fluid dynamic, thermal systems or robotics and control systems (just to name a few). There are different ways to define and approach pH-DAEs, such as using relations [4] or Dirac structures [24]. Here, we use an operator formulation, as in [13]. To be more specific, we consider

4 $$\begin{aligned} \frac{\hbox {d}}{{\hbox {d}}t}Ex(t)=AQx(t),\quad t\ge 0 \end{aligned}$$

with $E,Q\in L(\mathcal {X},\mathcal {Z})$, *Q* invertible, $A:\mathcal {D}(A)\subseteq \mathcal {Z}\rightarrow \mathcal {Z}$ closed, densely defined and dissipative and $E^*Q \ge 0$ non-negative, i.e. $\langle E^*Q x,x\rangle \ge 0$ for all $x\in \mathcal {X}$. The Hamiltonian of the pH-DAE (4) is given by $\langle E^*Q x,x\rangle$. Additionally to this, we will assume that $\textrm{ran}\, E$ is closed. This guarantees the dissipation of the energy

5 $$\begin{aligned} \frac{\hbox {d}}{{\hbox {d}}t}\langle E^*Q x(t), x(t)\rangle \le 0, \end{aligned}$$

along all classical solutions of (4).

The paper is organized as follows. In Sect. 2 a set of initial conditions for which (1) has a solution is characterized. In Sect. 3 the radiality index is defined and it is shown that if $\textrm{ran}\, E$ is closed, existence of this index implies existence of a Weierstraß form. Several new results relating the radiality index and the degree of nilpotency are also obtained. Furthermore, provided that the complex resolvent index exists, and the Weierstraß form exists with densely defined $A_1$, then $A_1$ generates an integrated semigroup.

In Sect. 4 port-Hamiltonian DAEs are formally described and their dissipativity is proven. Solvability of port-Hamiltonian DAEs is studied in Sect. 5. With the aid of the results earlier in the paper, it is shown that pH-DAEs have complex resolvent index at most 3. This extends a result that is known in finite dimensions to the infinite-dimensional setting. Existence of solutions to pH-DAEs is then obtained, along with generation of an integrated semigroup.

## Existence of solutions on a subspace

Throughout this article, let $\mathcal {X}$ and $\mathcal {Z}$ be Hilbert spaces, and, if not mentioned otherwise, $A:\mathcal {D}(A)\subseteq \mathcal {X}\rightarrow \mathcal {Z}$ is a closed and densely defined operator and $E\in L(\mathcal {X},\mathcal {Z})$. In this section, we prove that if that the complex resolvent index exists, solutions exist on a subspace. We first recall the definition of the complex resolvent index, see [2, 22].

### Definition 2.1

The *(complex) resolvent index* of (1) is the smallest number $p=p_{\textrm{res}}^{(E,A)}\in \mathbb {N}$ $(p=p_{\textrm{c,res}}^{(E,A)})$, such that there exists a $\omega \in \mathbb {R}$, $C>0$ with $(\omega ,\infty )\subseteq \rho (E,A)$ $(\mathbb {C}_{\textrm{Re}>\omega }\subseteq \rho (E,A))$ and

6 $$\begin{aligned} \Vert (\lambda E-A)^{-1} \Vert \le C\vert \lambda \vert ^{p-1},\quad \lambda \in (\omega ,\infty ) \; (\lambda \in \mathbb {C}_{\textrm{Re}>\omega }). \end{aligned}$$

In the following we determine a set of initial conditions for which the differential algebraic Eq. (1) has a classical solution.

In this context, we say that $x:\mathbb {R}_{\ge 0}\rightarrow \mathcal {X}$ is a *classical solution* of (1) for an initial condition $x_0\in \mathcal {X}$, if $x(\cdot )$ is continuous on $\mathbb {R}_{\ge 0}$, $Ex(\cdot )$ is continuously differentiable on $\mathbb {R}_{>0}$, $x(t)\in \mathcal {D}(A)$ for all $t\ge 0$, $x(0)=x_0$ and (1) holds, and we say that $x:\mathbb {R}_{\ge 0}\rightarrow \mathcal {X}$ is a *mild solution*, if $Ex(\cdot )$ is continuous, and for almost all $t\ge 0$, it holds $\int _0^t x(s)\,\textrm{d}s \in \mathcal {D}(A)$ with

$$\begin{aligned} Ex(t)- Ex_0 = A\int _0^t x(s)\,\textrm{d}s. \end{aligned}$$

The following theorem is a generalization of the solution theory of the abstract Cauchy problem $\frac{d}{dt} x(t)=Ax(t)$, $x(0)=x_0$ and extends the proofs of [10, p. 34] and [16, Ch. 4, Thm. 1.2] to the DAE case with the help of pseudo-resolvents.

### Theorem 2.2

Assume that (*E*, *A*) has a complex resolvent index. Then, for every $x_0 \in \textrm{ran}\, ((\mu E-A)^{-1} E)^{p_{\textrm{c,res}}^{(E,A)}+2}$ there exists a unique classical solution $x:\mathbb {R}_{\ge 0}\rightarrow \mathcal {X}$ of (1).

### Proof

*Existence*: Let $p =p_{\textrm{c,res}}^{(E,A)}+2$ and, for the sake of simplicity, define $R(\mu ) :=((\mu E-A)^{-1} E)$ for $\mu \in \rho (E,A)$. By assumption, the complex resolvent index exists. Thus, there are $\omega ,C >0$, such that

7 $$\begin{aligned} \left\| R(\lambda ) \right\| \le C \left| \lambda \right| ^{p_{\textrm{c,res}}^{(E,A)}-1} \left\| E \right\| , \quad \lambda \in \mathbb {C}_{\textrm{Re} \ge \omega }. \end{aligned}$$

Let $x_0 \in \textrm{ran}\, R(\mu )^p$. Thus, there exist a $z_0\in \mathcal {X}$ and a $\mu \in \mathbb {C}_{\textrm{Re} > \omega }$ such that $x_0= (-1)^{p-1} R(\mu )^{p}z_0$ and therefore

8 $$\begin{aligned} \left\| \frac{R(\lambda )}{(\lambda -\mu )^p}\right\| \le {\tilde{C}} \frac{\left| \lambda \right| ^{p-3}}{\left| \lambda -\mu \right| ^p}, \end{aligned}$$

for a ${\tilde{C}}>0$ and for all $\lambda \ne \mu$. Thus, the integral of $\textrm{e}^{\lambda t}\frac{R(\lambda )z_0}{(\lambda -\mu )^p}$ along the imaginary axis at $\textrm{Re} \,\lambda = \omega$ exists and, especially, the function

9 $$\begin{aligned} x(t):=\frac{(-1)^p}{2\pi i} \int _{\omega -i\infty }^{\omega +i\infty } \textrm{e}^{\lambda t} \frac{R(\lambda )z_0}{(\lambda -\mu )^p}\,\textrm{d}\lambda , \quad t\ge 0, \end{aligned}$$

is continuously differentiable. Since *E* is bounded, the function *Ex*(*t*) becomes differentiable as well and

10 $$\begin{aligned} \frac{d}{dt}Ex(t)&= \frac{(-1)^p}{2\pi i} \int _{\omega -i\infty }^{\omega +i\infty } \lambda \textrm{e}^{\lambda t} \frac{E R(\lambda )z_0}{(\lambda -\mu )^p}\,\textrm{d}\lambda \end{aligned}$$

11 $$\begin{aligned}&= \frac{(-1)^p}{2\pi i} \int _{\omega -i\infty }^{\omega +i\infty } \textrm{e}^{\lambda t} \frac{AR(\lambda )z_0}{(\lambda -\mu )^p}\,\textrm{d}\lambda + \underbrace{\frac{(-1)^p}{2\pi i} \int _{\omega -i\infty }^{\omega +i\infty } \textrm{e}^{\lambda t} \frac{Ez_0}{(\lambda -\mu )^p}\,\textrm{d}\lambda }_{=0} = A x(t). \end{aligned}$$

Here, we used that $\frac{Ez_0}{(\lambda -\mu )^p}$ converges uniformly to 0 for $\lambda \rightarrow \infty$ and Jordan’s lemma to show that the second integral in (11) vanishes. Furthermore,

$$\begin{aligned} x(0) = \frac{(-1)^{p}}{2\pi i} \int _{\omega -i \infty }^{\omega + i \infty } \frac{R(\lambda )z_0}{(\lambda -\mu )^p}\,\textrm{d}\lambda . \end{aligned}$$

Using again Jordan’s lemma and, additionally, the residue theorem, we derive

$$\begin{aligned} x(0) = \frac{(-1)^{p-1}}{(p-1)!} \lim _{\lambda \rightarrow \mu } \frac{d^{p-1}}{d \lambda ^{p-1}} R(\lambda ) z_0 = (-1)^{p-1} R(\mu )^p z_0 = x_0. \end{aligned}$$

Here, we used $\frac{d}{d\lambda } R(\lambda )^n z_0 = - n R(\lambda )^{n+1} z_0$ for all $n \in \mathbb {N}$ and $\lambda \in \rho (E,A)$ ([19, Lem. 2.4.1]). Thus, *x* is a classical solution of (1).

*Uniqueness:* We assume that *x* is a solution with $x(0) = 0$ and prove that $x({t}) = 0$ for all $t\ge 0$. Since *x* is a solution of (1), we deduce

$$\begin{aligned} \frac{d}{dt} R(\lambda ) x(t) = (\lambda E -A)^{-1} A x(t) = \lambda R(\lambda ) x(t) - x(t), \end{aligned}$$

for an $\lambda \in \rho (E,A)$. The solution of this equation can be written as

12 $$\begin{aligned} R(\lambda ) x(t) = \textrm{e}^{\lambda t } x(0) - \int _0^t \textrm{e}^{\lambda (t-\tau )}x(\tau )\,\textrm{d}\tau = -\int _0^t \textrm{e}^{\lambda (t-\tau )}x(\tau )\,\textrm{d}\tau . \end{aligned}$$

Let $\sigma >0$. Inequality (7) then implies

13 $$\begin{aligned} \lim _{\textrm{Re} \lambda \rightarrow \infty } \textrm{e}^{-\sigma \lambda } \left\| R(\lambda ) \right\| = 0. \end{aligned}$$

By (12), (13) and the fact that a solution *x* is bounded on [0, *t*] we conclude

$$\begin{aligned} \lim _{\textrm{Re} \lambda \rightarrow \infty } \bigg \Vert \int _0^{t-\sigma }&\textrm{e}^{\lambda (t-\sigma -\tau )} x({\tau })\,\textrm{d}\tau \bigg \Vert \\&= \lim _{\textrm{Re} \lambda \rightarrow \infty } \textrm{e}^{-\sigma \textrm{Re} \lambda } \left\| \int _0^{t-\sigma } \textrm{e}^{\lambda (t-\tau )}x({\tau })\,\textrm{d}\tau \right\| \\&\le \lim _{\textrm{Re} \lambda \rightarrow \infty } \textrm{e}^{-\sigma \textrm{Re}\lambda } \bigg \Vert \underbrace{\int _0^t \textrm{e}^{\lambda (t-\tau )}x({\tau })\,\textrm{d}\tau }_{=R(\lambda ) x(t)} \bigg \Vert + \underbrace{\textrm{e}^{-\sigma \textrm{Re}\lambda } \left\| \int _{t-\sigma }^t \textrm{e}^{\lambda (t-\tau )}x({\tau })\,\textrm{d}\tau \right\| }_{\rightarrow 0} = 0. \end{aligned}$$

Thus,

$$\begin{aligned} \lim _{\textrm{Re} \lambda \rightarrow \infty } \int _0^{t-\sigma } \textrm{e}^{\lambda (t-\sigma -\tau )} x(\tau )\,\textrm{d}\tau = 0 \end{aligned}$$

and by [16, Ch. 4, Lem. 1.1] $x(\tau ) = 0$ for all $0\le \tau \le t-\sigma$. Since $\sigma$ and *t* were arbitrary, we deduce $x(\tau ) = 0$ for all $\tau \ge 0$. $\square$

### Remark 2.3

- Of course it is also possible to formulate the initial condition $x(0)=x_0$ of (1) as
  $$\begin{aligned} {\left\{ \begin{array}{ll} \frac{d}{dt}Ex(t) = Ax(t),\quad t\ge 0,\\ Ex(0) = x_0. \end{array}\right. } \end{aligned}$$
  Accordingly, one must assume $x_0\in E(\textrm{ran}\, ((\mu E-A)^{-1} E)^{p_{\textrm{c,res}}^{(E,A)}+2})$ in Theorem 2.2.
- The term $p=p_{\textrm{c,res}}^{(E,A)}+2$ in the proof of Theorem 2.2 is chosen in such a way that $\frac{R(\lambda )}{(\lambda -\mu )^p}$ falls quickly enough, such that the integral in (9) not only exists; the function *x*(*t*) also becomes continuously differentiable. This means that the function *x* in (9) is only continuous, if $x_0 \in \textrm{ran}\, ((\mu E-A)^{-1} E)^{p_{\textrm{c,res}}^{(E,A)}+1}$. However, in this case *x* is a mild solution of (1), as one can compute
  $$\begin{aligned} Ex(t)- Ex(0)&= -\frac{1}{2\pi i}\int _{\omega -i \infty }^{\omega + i \infty } \underbrace{(\textrm{e}^{\lambda t }-1)}_{=\int _0^t \lambda \textrm{e}^{\lambda s}\,\textrm{d}s} \frac{E R(\lambda )z_0}{(\lambda -\mu )^p}\,\textrm{d}\lambda \\&= \int _0^t \bigg ( -\frac{1}{2\pi i}\int _{\omega -i \infty }^{\omega + i \infty } \lambda \textrm{e}^{\lambda s} \frac{{E}R(\lambda )z_0}{(\lambda -\mu )^p}\,\textrm{d}\lambda \bigg )\,\textrm{d}s\\&= \int _0^t \bigg ( -\frac{1}{2\pi i} \int _{\omega -i\infty }^{\omega +i\infty } \textrm{e}^{\lambda s} \frac{AR(\lambda )z_0}{(\lambda -\mu )^p}\,\textrm{d}\lambda - \underbrace{\frac{1}{2\pi i} \int _{\omega -i\infty }^{\omega +i\infty } \textrm{e}^{\lambda s} \frac{Ez_0}{(\lambda -\mu )^p}\,\textrm{d}\lambda }_{=0} \bigg ) \,\textrm{d}s \\&= \int _0^t Ax(s)\,\textrm{d}s = A \int _0^t x(s)\,\textrm{d}s. \end{aligned}$$
- In [22, Thm. 5.7] the set of initial values
  14 $$\begin{aligned} U:=\{x_0 \in \mathcal {X} \, \vert \, \exists x:\mathbb {R}_{\ge 0}\rightarrow \mathcal {X} \text { mild solution}\} \end{aligned}$$
  was analysed in more detail. To be more precise, it was shown that $E^{-1} A$ generates a $C_0$-semigroup on $\overline{\textrm{ran}\, ((\mu E-A)^{-1} E)^{p_{\textrm{c,res}}^{(E,A)}+2}}$ if and only if *U* is closed and for each $x_0 \in U$ the mild solution is unique. The assumption that *U* is closed is a bit restrictive and it also requires information about *U*.
- In [5, Sect. 8] the existence of solutions on the subspace $\overline{\textrm{ran}\, R(\lambda )^k}$, for $R(\lambda ):=(\lambda E-A)^{-1} E$, has been studied under slightly stronger conditions compared to the ones in Theorem 2.2. More specifically, under the assumption that there exists $k\in \mathbb {N}$ with $k\ge 1$, $\omega \in \mathbb {R}$ and $M>0$, such that $(\omega ,\infty )\subseteq \rho (E,A)$ and
  $$\begin{aligned} \left\| R(\lambda )x\right\| \le \frac{M}{\lambda -\omega } \left\| x\right\| , \quad \forall \lambda >\omega , \; x\in \textrm{ran}\, R(\omega )^{k-1} \end{aligned}$$
  the following operator $A_R$ was defined through its graph
  $$\begin{aligned} \mathrm{{graph}}\, A_R = \{ (R(\lambda ) x, x+\lambda R(\lambda )x)\, \vert \, x\in \overline{\textrm{ran}\, R(\lambda )^k}\}. \end{aligned}$$
  Then, under the assumption that $A_R$ generates a $C_0$-semigroup, it was shown that for every $x_0\in \overline{\textrm{ran}\, R(\lambda )^k}$ there exists a unique mild solution of (1) and for every $x_0 \in R(\lambda )\overline{\textrm{ran}\, R(\lambda )^k}$ there exists a classical solution of (1). As it was remarked by the authors, the generation of the $C_0$-semigroup can be achieved if
  $$\begin{aligned} \left\| \lambda R(\lambda ) x\right\| \le M \left\| x\right\| \quad \forall \lambda \in \mathbb {C}_{\ge \omega }, \; x\in \textrm{ran}\, R(\omega )^k \end{aligned}$$
  holds, which is a slightly stronger assumption compared to the complex resolvent index. In the latter case, we only have
  $$\begin{aligned} \left\| \lambda R(\lambda ) R(\mu )^k x \right\| \le M \left\| x\right\| \quad \forall \lambda \in \mathbb {C}_{\ge \omega },\; x\in \mathcal {X}, \end{aligned}$$
  for $$k=p_{\textrm{c,res}}^{(E,A)}$$.

## Sufficient condition for a Weierstraß form

In this section we start with the analysis of a sufficient condition for the existence of a Weierstraß form. To do that, we recall the definition of the radiality index [2] or, more generally, of the radiality [19].

We call two differential-algebraic equations $\frac{d}{dt}Ex=Ax$ and $\frac{d}{dt}{\tilde{E}} {\tilde{x}}={\tilde{A}}{\tilde{x}}$ defined on Hilbert spaces $\mathcal {X}$, $\mathcal {Z}$ and $\tilde{\mathcal {X}}$, $\tilde{\mathcal {Z}}$ *equivalent*, denoted by $(E,A)\sim ({\tilde{E}},{\tilde{A}})$, if there are two bounded isomorphisms $P:\mathcal {X}\rightarrow \tilde{\mathcal {X}}$, $Q:\mathcal {Z}\rightarrow \tilde{\mathcal {Z}}$, such that $E=Q^{-1} {\tilde{E}} P$ and $A=Q^{-1} {\tilde{A}} P$. Based on that, we define the notion of a Weierstraß form as follows.

### Definition 3.1

The system (1) has a *Weierstraß form*, if there exists a Hilbert space $\mathcal {Y}=\mathcal {Y}^1\oplus \mathcal {Y}^2$, such that

15 $$\begin{aligned} (E,A) \sim \left( \begin{bmatrix} I_{\mathcal {Y}^1} &  0\\ 0 &  N \end{bmatrix}, \begin{bmatrix} A_1 &  0 \\ 0 &  I_{\mathcal {Y}^2} \end{bmatrix}\right) , \end{aligned}$$

where $N:\mathcal {Y}^2\rightarrow \mathcal {Y}^2$ is a nilpotent operator, $A_1:\mathcal {D}(A_1)\subseteq \mathcal {Y}^1\rightarrow \mathcal {Y}^1$ is a linear operator, and $I_{\mathcal {Y}^i}$ indicates the identity operator on the associated subspace $\mathcal {Y}^i$, $i=1,2$. Furthermore, the nilpotency degree of *N* is known as the *nilpotency index* $p_{\textrm{nilp}}^{(E,A)}$ [2, Sect. 2].

### Definition 3.2

- The system (1) has a *(complex) radiality index*, if there exists a $p=p_{\textrm{rad}}^{(E,A)}\in \mathbb {N}$ $(p=p_{\textrm{c, rad}}^{(E,A)}\in \mathbb {N})$ and $\omega , C>0$, such that $(\omega , \infty )\subseteq \rho (E,A)$ ($\mathbb {C}_{\textrm{Re}>\omega }\subseteq \rho (E,A)$) and
  16 $$\begin{aligned} \begin{aligned} \left\| \left( (\lambda _0E-A)^{-1} E\cdot \cdots \cdot (\lambda _p E-A)^{-1} E\right) ^n\right\| \le \frac{C}{\prod _{k=0}^{p}\vert \lambda _k-\omega \vert ^n},\\ \left\| \left( E(\lambda _0E-A)^{-1} \cdot \cdots \cdot E(\lambda _p E-A)^{-1}\right) ^n \right\| \le \frac{C}{\prod _{k=0}^{p}\vert \lambda _k-\omega \vert ^n}, \end{aligned} \end{aligned}$$
  holds for all $\lambda _0,\ldots ,\lambda _{p}\in (\omega ,\infty )$ $(\lambda _0,\ldots ,\lambda _{p}\in \mathbb {C}_{\textrm{Re}>\omega })$ and $n=1$.
- The system (1) is *p*-*radial*, if it has radiality index *p* and (16) holds for all $n\in \mathbb {N}$.

### Theorem 3.3

Let $E\in L(\mathcal {X},\mathcal {Z})$ with closed range and $A:\mathcal {D}(A)\subseteq \mathcal {X}\rightarrow \mathcal {Z}$ be a closed and densely defined operator. If (*E*, *A*) has a radiality index, then it also has a Weierstraß form, which is unique up to isomorphisms.

### Proof

Let $p=p_{\textrm{rad}}^{(E,A)}$ denote the radiality index. For arbitrary $\lambda _0,\ldots ,\lambda _p \in \rho (E,A)$ define

$$\begin{aligned} \mathcal {X}^1&= \overline{\textrm{ran}\, (\lambda _0 E-A)^{-1} E \cdot \cdots \cdot (\lambda _p E-A)^{-1} E }^{\Vert \cdot \Vert _{\mathcal {X}}}, \\ \mathcal {X}^2&= \textrm{ker}\, (\lambda _0 E-A)^{-1} E \cdot \cdots \cdot (\lambda _p E-A)^{-1} E,\\ \mathcal {Z}^1&= \overline{\textrm{ran}\, E(\lambda _0 E-A)^{-1} \cdot \cdots \cdot E (\lambda _p E-A)^{-1}}^{\Vert \cdot \Vert _{\mathcal {Z}}}, \\ \mathcal {Z}^2&= \textrm{ker}\, E (\lambda _0 E-A)^{-1} \cdot \cdots \cdot E(\lambda _p E-A)^{-1}. \end{aligned}$$

By [19, Sects. 2.1, 2.2 and 2.5] these spaces are independent of the choice of $\lambda _i$, $i\in \{0,\ldots ,p\}$,

$$\begin{aligned} E_2&:=E\vert _{\mathcal {X}^2} \in L(\mathcal {X}^2,\mathcal {Z}^2),\\ A_2&:=A\vert _{\mathcal {D}(A_2)} :\mathcal {D}(A_2)\subseteq \mathcal {X}^2\rightarrow \mathcal {Z}^2,\\ \mathcal {D}(A_2)&:=\mathcal {D}(A)\cap \mathcal {X}^2 \end{aligned}$$

is boundedly invertible operator with $A_2^{-1} \in L(\mathcal {Z}^2,\mathcal {X}^2)$, such that $A_2^{-1} E_2\in L(\mathcal {X}^2)$ and $E_2A_2^{-1}\in L(\mathcal {Z}^2)$ are nilpotent with degree less or equal $p+1$. Furthermore, there are two projections, namely

$$\begin{aligned} P:\mathcal {X}\rightarrow \mathcal {X}, \quad x\mapsto \lim _{\lambda \rightarrow \infty } (\lambda (\lambda E-A)^{-1} E)^{p+1} x \end{aligned}$$

onto $\mathcal {X}^1$ with $\textrm{ker}\, P =\mathcal {X}^2$, $\textrm{ran}\, P =\mathcal {X}^1$, $Px_1=x_1$ for all $x_1\in \mathcal {X}^1$ and

$$\begin{aligned} R:\mathcal {Z}\rightarrow \mathcal {Z}, \quad z\mapsto \lim _{\lambda \rightarrow \infty } (\lambda E (\lambda E-A)^{-1} )^{p+1} z \end{aligned}$$

onto $\mathcal {Z}^1$ with $\textrm{ker}\, R = \mathcal {Z}^2$, $\textrm{ran}\, R = \mathcal {Z}^1$, $Rz_1=z_1$ for all $z_1\in \mathcal {Z}^1$, such that $\mathcal {X}=\mathcal {X}^1\oplus \mathcal {X}^2$ and $\mathcal {Z}=\mathcal {Z}^1\oplus \mathcal {Z}^2$. Furthermore, $(\lambda E-A)^{-1} E x\in \mathcal {D}(A)$ for all $x\in \mathcal {D}(A)$ and thus, since *A* is closed, $Px\in \mathcal {D}(A)$. Hence,

17 $$\begin{aligned} \begin{aligned} APx&= A \lim _{\lambda \rightarrow \infty } (\lambda (\lambda E-A)^{-1} E)^{p+1}x\\&=\lim _{\lambda \rightarrow \infty } A (\lambda (\lambda E-A)^{-1} E)^{p+1}x\\&= \lim _{\lambda \rightarrow \infty } (E\lambda (\lambda E-A)^{-1})^{p+1} Ax\\&= RAx. \end{aligned} \end{aligned}$$

Since *E* is bounded we derive

18 $$\begin{aligned} \begin{aligned} EPx&= E \lim _{\lambda \rightarrow \infty } (\lambda (\lambda E-A)^{-1} E)^{p+1}x\\&=\lim _{\lambda \rightarrow \infty } E (\lambda (\lambda E-A)^{-1} E)^{p+1}x\\&= \lim _{\lambda \rightarrow \infty } (E\lambda (\lambda E-A)^{-1})^{p+1} Ex\\&= REx \end{aligned} \end{aligned}$$

for all $x\in \mathcal {X}$. Now, we need to show that

$$\begin{aligned} E_1&:=E\vert _{\mathcal {X}^1}\in L(\mathcal {X}^1, \mathcal {Z}^1)\\ \end{aligned}$$

and

$$\begin{aligned} A_i&:=A\vert _{\mathcal {D}(A_i)}:\mathcal {D}(A_i)\subseteq \mathcal {X}^i\rightarrow \mathcal {Z}^i \end{aligned}$$

is closed and densely defined, whereby $\mathcal {D}(A_i):=\mathcal {D}(A)\cap \mathcal {Z}^i$, $i\in \{1,2\}$. This was already proved for $p=0$ in [7, Prop. 2.5] and under stronger radiality assumptions in [19, Cor. 2.5.2, Thm. 2.5.3]. Let $x\in \mathcal {X}^1$. Since $\mathcal {X}=\mathcal {X}^1\oplus \mathcal {X}^2$, $x\in \mathcal {X}^1$ if and only if $Px=x$. By (18) we conclude

$$\begin{aligned} Ex=EPx=REx \in \mathcal {Z}^1 \end{aligned}$$

and, since *E* is bounded, the same yields for $E_1$. Analogously, using (17) we have $Ax=APx=RAx\in \mathcal {Z}^1$ for all $x\in \mathcal {D}(A_1)$. Now, let $x\in \mathcal {X}^1$. Thus, there exists a sequence $(x_n)_n\subseteq \mathcal {D}(A)$ with $x_n\rightarrow x$ for $n\rightarrow \infty$. Since $Px_n\in \mathcal {D}(A_1)$ and $Px_n\rightarrow Px=x$ for $n\rightarrow \infty$, one has $\overline{\mathcal {D}(A_1)}=\mathcal {X}^1$. Similarly, using $I-P$ one can show $\overline{\mathcal {D}(A_2)}=\mathcal {X}^2$. Since *A* is closed and $A_i(\mathcal {D}(A_i))\subseteq \mathcal {X}^i$, $A_i$ is closed as well, $i\in \{1,2\}$.

What is left to show is that $E_1$ is boundedly invertible. Since $E_1$ is bounded, $\textrm{ker}\, E_1\subseteq \textrm{ker}\, E\subseteq \mathcal {X}^2$ and $\mathcal {X}=\mathcal {X}^1\oplus \mathcal {X}^2$, it follows that $E_1$ is injective. What remains to show is the surjectivity of *E*, because then the assertion follows from the closed graph theorem. Since the space $\mathcal {Z}^1$ does not depend on the choice of the $\lambda _i$, we can choose a $\lambda \in \rho (E,A)$, such that $\mathcal {Z}^1=\overline{\textrm{ran}\, (E(\lambda E-A)^{-1} )^{p+2}}^{\Vert \cdot \Vert _{\mathcal {Z}}}$ [19, Lem. 2.2.8]. Let $y\in \textrm{ran}\, (E(\lambda E-A)^{-1})^{p+2}$. Then there exists a $z\in \mathcal {Z}$ with

$$\begin{aligned} y = (E(\lambda E-A)^{-1} )^{p+2}z = E ((\lambda E-A)^{-1} E)^{p+1} (\lambda E-A)^{-1} z \end{aligned}$$

and thus

$$\begin{aligned} \textrm{ran}\, (E(\lambda E-A)^{-1})^{p+2} \subseteq E(\textrm{ran}\, ((\lambda E-A)^{-1} E)^{p+1})\subseteq E(\mathcal {X}^1). \end{aligned}$$

Since $\textrm{ran}\, E$ is closed, the same yields for $E(\mathcal {X}^1) = \textrm{ran}\, E_1 = \mathcal {Z}^1\cap \textrm{ran}\, E$. Thus, the closure of $\textrm{ran}\,(E(\lambda E-A)^{-1})^{p+2}$ is still a subset of $E(\mathcal {X}^1)$. Since $\textrm{ran}\, E_1\subseteq \mathcal {Z}^1 = \overline{\textrm{ran}\, (E(\lambda E-A)^{-1} )^{p+2}}^{\Vert \cdot \Vert _{\mathcal {Z}}}$ one obtains the surjectivity of *E*. Define

$$\begin{aligned} {\tilde{P}} = \begin{bmatrix}P\\ I_{\mathcal {X}}-P\end{bmatrix} \in L(\mathcal {X},\mathcal {X}^1\times \mathcal {X}^2), \quad {\tilde{R}}=\begin{bmatrix}R\\ I_{\mathcal {Z}}-R\end{bmatrix}\in L(\mathcal {Z},\mathcal {Z}^1\times \mathcal {Z}^2). \end{aligned}$$

Then,

$$\begin{aligned} {\tilde{P}} ^{-1} = \begin{bmatrix}I_{\mathcal {X}^1}&I_{\mathcal {X}^2}\end{bmatrix} \in L(\mathcal {X}^1\times \mathcal {X}^2, \mathcal {X}), \quad {\tilde{R}}^{-1} = \begin{bmatrix}I_{\mathcal {Z}^1}&I_{\mathcal {Z}^2}\end{bmatrix} \in L(\mathcal {Z}^1\times \mathcal {Z}^2, \mathcal {Z}). \end{aligned}$$

Thus,

19a $$\begin{aligned} (E,A)&\sim ({\tilde{R}} E {\tilde{P}} ^{-1}, {\tilde{R}} A {\tilde{P}} ^{-1})\end{aligned}$$

19b $$\begin{aligned}&\sim \left( \begin{bmatrix} E_1 &  0 \\ 0 &  E_2\end{bmatrix}, \begin{bmatrix} A_1 &  0 \\ 0 &  A_2\end{bmatrix} \right) \end{aligned}$$

19c $$\begin{aligned}&\sim \left( \begin{bmatrix} I_{\mathcal {Z}^1} &  0 \\ 0 &  E_2A_2^{-1}\end{bmatrix}, \begin{bmatrix} A_1E_1^{-1} &  0 \\ 0 &  I_{\mathcal {Z}^2}\end{bmatrix} \right) . \end{aligned}$$

The uniqueness of the Weierstraß form follows from the uniqueness of the nilpotency index [2, Prop. 6.2], which exists since the nilpotency index of (*E*, *A*) is at most $p_{\textrm{rad}}^{(E,A)}+1$ [2, Prop. 6.3]. $\square$

### Remark 3.4

With (19c) one can rewrite the DAE $\frac{d}{dt}Ex=Ax$ as follows

20 $$\begin{aligned} \frac{d}{dt}\begin{bmatrix} I_{\mathcal {Z}^1} &  0 \\ 0 &  E_2A_2^{-1}\end{bmatrix} \begin{bmatrix}z_1(t) \\ z_2(t)\end{bmatrix} = \begin{bmatrix} A_1E_1^{-1} &  0 \\ 0 &  I_{\mathcal {Z}^2}\end{bmatrix} \begin{bmatrix}z_1(t) \\ z_2(t)\end{bmatrix}, \end{aligned}$$

on $$\mathcal {Z}^1\times \mathcal {Z}^2$$.

Further, one can also show that

$$\begin{aligned} (E,A)\sim \left( \begin{bmatrix} I_{\mathcal {X}^1} &  0 \\ 0 &  A_2^{-1} E_2 \end{bmatrix}, \begin{bmatrix} E_1^{-1} A_1 &  0 \\ 0 &  I_{\mathcal {X}^2}\end{bmatrix}\right) \end{aligned}$$

and rewrite the DAE $E\frac{d}{dt}x = Ax$ in the following way

21 $$\begin{aligned} \begin{bmatrix} I_{\mathcal {X}^1} &  0 \\ 0 &  A_2^{-1} E_2\end{bmatrix} \frac{d}{dt} \begin{bmatrix}x_1(t) \\ x_2(t)\end{bmatrix}&= \begin{bmatrix} E_1^{-1} A_1 &  0 \\ 0 &  I_{\mathcal {X}^2}\end{bmatrix} \begin{bmatrix}x_1(t) \\ x_2(t)\end{bmatrix}, \end{aligned}$$

on $$\mathcal {X}^1\times \mathcal {X}^2$$.

In the following, we will recall and generalize an example from [2, Ex. 6.5], which shows that the radiality index can be greater than 0.

### Example 3.5

Let $\mathcal {W}$ be a Hilbert space and $(A_0, \mathcal {D}(A_0))$ generate a $C_0$-semigroup on $\mathcal {W}$. For $b\in \mathcal {D}(A_0)$ and $c \in \mathcal {D}(A_0^*)$ with $\langle b, c\rangle \ne 0$ define the operators $B_u=bu$, where $u\in \mathbb {C}$ and $Cz=\langle c,z\rangle$ for any $z\in \mathcal {W}$. We define the following DAE on $\mathcal {Z}=\mathcal {W}\times \mathbb {C}$ by

22 $$\begin{aligned} \frac{d}{dt}\underbrace{\begin{bmatrix} I &  0\\ 0 &  0\end{bmatrix}}_{=:E} x(t) = \underbrace{\begin{bmatrix} A_0 &  B\\ C &  0\end{bmatrix}}_{=:A} x(t), \quad t\ge 0. \end{aligned}$$

In [2] it was already proven that the resolvent index is at most 2 and for specific $A_0$, *B* and *C* it was also shown that the radiality index is 1. We want to generalize the last statement a bit. To do this, we look again at the left- and right-*E* resolvents.

We want to show that the radiality index exists by using the Weierstraß form. Define $Q_b z = z-\frac{\langle c,z\rangle }{\langle c, b\rangle } b$. Then, $Q_b b=0$, $\textrm{ran}\, Q_b=\textrm{ker}\, C$ and $\mathcal {W}=\mathcal {W}^1\oplus \mathcal {W}^2 :=\textrm{ker}\, C \oplus \textrm{span}(b)$. For more concise notation, we additionally define ${\tilde{C}} = \frac{1}{\langle c,b\rangle }{C}$, ${\tilde{B}} = \frac{1}{\langle c,b\rangle } B$ and $K:={\tilde{C}} A_0$. Note that $Kz:={\tilde{C}} A_0 z = \frac{1}{\langle c,b\rangle } \langle c, A_0 z \rangle = \frac{1}{\langle c,b\rangle } \langle A_0^*c, z \rangle$ holds for all $z\in W$ and therefore *K* is a bounded operator on $\mathcal {W}$. Thus, we can define the isomorphisms $U:\mathcal {W} \times \mathbb {C} \rightarrow \mathcal {W}^1 \times \mathcal {W}^2\times \mathbb {C}$ and $V:\mathcal {W}^1\times \mathcal {W}^2\times \mathbb {C}\rightarrow \mathcal {W}\times \mathbb {C}$ via

$$\begin{aligned}&U:=\begin{bmatrix} Q_b &  -Q_b A_0 {\tilde{B}} \\ 0 &  {\tilde{B}} \\ {\tilde{C}} &  -K{\tilde{B}}\end{bmatrix},&V:=\begin{bmatrix} I_{\mathcal {W}^1} &  I_{\mathcal {W}^2} &  0\\ -K &  0 &  1\end{bmatrix}, \end{aligned}$$

with inverses

$$\begin{aligned} U^{-1}&:=\begin{bmatrix} I_{\mathcal {W}^1} &  A_0 &  B\\ 0 &  C &  0\end{bmatrix},&V^{-1}&:=\begin{bmatrix} Q_b &  0\\ I_{\mathcal {W}}-Q_b &  0\\ KQ_b &  1\end{bmatrix}. \end{aligned}$$

These mappings applied to (22) we derive

$$\begin{aligned} {\tilde{E}} :=U \begin{bmatrix} I_{\mathcal {W}} &  0 \\ 0 &  0\end{bmatrix} V= \begin{bmatrix} I_{\mathcal {W}^1} &  0\\ 0 &  N \end{bmatrix}, \qquad {\tilde{A}} :=U \begin{bmatrix} A_0 &  B\\ C &  0\end{bmatrix} V = \begin{bmatrix} Q_b A_0 &  0 \\ 0 &  I_{\mathcal {W}^2\times \mathbb {C}} \end{bmatrix}, \end{aligned}$$

with $$N=\begin{bmatrix} 0 &  0 \\ {\tilde{C}} &  0 \end{bmatrix}$$. Since $A_0$ is a generator of a $C_0$-semigroup, $Q_b A_0 z = A_0 z - \frac{\langle c, A_0 z\rangle }{\langle c,b\rangle } b = A_0 z - BK z$, $z\in \mathcal {W}^1$ and *BK* is bounded, $Q_bA_0$ also generates a $C_0$-semigroup. Moreover, $({\tilde{E}}, {\tilde{A}})$ has a Weierstraß form as in (15) and together with the following Theorem 3.6 b) $({\tilde{E}}, {\tilde{A}})$ is 1-radial.

Further examples of DAEs with existing radiality index (such as the linearized Navier–Stokes equation) can be found in [3]. Next, the radiality index for DAEs in Weierstraß form gets investigated.

### Theorem 3.6

Assume that $$(E,A)= \left( \Big [{\begin{matrix} I_{\mathcal {Y}^1} &  0\\ 0 &  N \end{matrix}}\Big ], \Big [{\begin{matrix} A_1 &  0\\ 0 &  I_{\mathcal {Y}^2} \end{matrix}}\Big ]\right) $$, where $A_1$ and *N* are defined as in (15). Then

- (*E*, *A*) has radiality index $p_{\textrm{rad}}^{(E,A)}=p\in \mathbb {N}$ if and only if $(I_{\mathcal {Y}^1}, A_1)$ has radiality index $p_{\textrm{rad}}^{(E,A)}=p\in \mathbb {N}$ and *N* has nilpotency degree smaller or equal to $p+1$.
- If $A_1$ generates a $C_0$-semigroup and *N* has nilpotency degree smaller or equal to $p+1$, then (*E*, *A*) is *p*-radial and, especially, $p_{\textrm{rad}}^{(E,A)} = p$.

### Proof

Let *N* has nilpotency degree $k+1\in \mathbb {N}$ and $\lambda \in \rho (E,A)$. Then, $\lambda \in \rho (I_{\mathcal {Y}^1}, A_1)$. Conversely, if $\lambda \in \rho (I_{\mathcal {Y}^1}, A_1)$, then $(\lambda N-I_{\mathcal {Y}^2})^{-1} = -\sum _{l=0}^k (\lambda N)^l$. Thus,

23 $$\begin{aligned} \rho (E,A)=\rho (I_{\mathcal {Y}^1},A_1) = \rho (A_1). \end{aligned}$$

In particular, for $\lambda \in \rho (E,A)$ we obtain

24 $$\begin{aligned} (\lambda E- A)^{-1} E = \begin{bmatrix} (\lambda I_{\mathcal {Y}^1}-A_1)^{-1} &  0 \\ 0 &  (\lambda N-I_{\mathcal {Y}^2})^{-1} N \end{bmatrix} = {E(\lambda E- A)^{-1}} \end{aligned}$$

and

$$\begin{aligned} \prod _{l=0}^k (\lambda _l E-A)^{-1} E=\Big [{\begin{matrix} \prod _{l=0}^k(\lambda _l I_{\mathcal {Y}^1}-A_1)^{-1} &  0\\ 0 &  0 \end{matrix}}\Big ] \end{aligned}$$

for $\lambda _0,\ldots ,\lambda _p\in \rho ( E, A)$. In particular, we have

25 $$\begin{aligned} \left\| \prod _{l=0}^k(\lambda _l I_{\mathcal {Y}^1}-A_1)^{-1}\right\| = \left\| \prod _{l=0}^k (\lambda _l E-A)^{-1} E\right\| . \end{aligned}$$

Thus, Part a) follows directly from (23) and (25). To show Part b) assume that $A_1$ generates a $C_0$-semigroup and *N* has a nilpotency degree smaller or equal to $p+1$. Using the theorem of Hille-Yosida there exists $\omega , C>0$ such that $(\omega , \infty )\subseteq \rho (I_{\mathcal {Y}^1}, A_1) = \rho (A_1)$ and $\Vert ((\lambda I_{\mathcal {Y}^1}-A_1)^{-1})^n\Vert \le \frac{C}{(\lambda -\omega )^n}$ for all $\lambda \ge \omega$, $n\in \mathbb {N}$. Together with (24) and (25) we have

$$\begin{aligned} \left\| \left( \prod _{l=0}^p (\lambda _l E-A)^{-1} E\right) ^n\right\| = \left\| \left( \prod _{l=0}^p E(\lambda _l E- A)^{-1} \right) ^n\right\| \le \frac{C^{p+1}}{\left( \prod _{l=0}^{p+1}\lambda _l-\omega \right) ^n} \end{aligned}$$

for all $\lambda _0,\ldots ,\lambda _{p+1}\ge \omega$. Thus, (*E*, *A*) is *p*-radial. $\square$

A direct consequence of Theorem 3.6 is the equality of different index terms introduced in [2]. Here, we will denote the differentiation index, the chain index and the perturbation index by $p_{\textrm{diff}}^{(E,A)}$, $p_{\textrm{chain}}^{(E,A)}$ and $p_{\textrm{pert}}^{(E,A)}$.

### Corollary 3.7

Assume that (*E*, *A*) has a Weierstraß form and nilpotency index $p_{\textrm{nilp}}^{(E,A)}$, i.e. $$(E,A)\sim \left( \Big [{\begin{matrix} I_{\mathcal {Y}^1} &  0\\ 0 &  N \end{matrix}}\Big ], \Big [{\begin{matrix} A_1 &  0\\ 0 &  I_{\mathcal {Y}^2} \end{matrix}}\Big ]\right) $$. If $A_1$ generates a $C_0$-semigroup on $\mathcal {Y}^1$, then

$$\begin{aligned} p_{\textrm{rad}}^{(E,A)}+1 = p_{\mathrm{res\phantom {d}}}^{(E,A)} = p_{\textrm{nilp}}^{(E,A)} = p_{\textrm{diff}}^{(E,A)} = p_{\textrm{chain}}^{(E,A)} = p_{\textrm{pert}}^{(E,A)}. \end{aligned}$$

### Proof

This follows from Theorem 3.6 and [2]. $\square$

Next, we want to investigate the connection between integrated semigroups and linear differential algebraic equations.

### Definition 3.8

A linear operator *A* on a Banach space $\mathcal {X}$ is the *generator of an* $(n-1)$-*times integrated semigroup* $(S(t))_{t\ge 0}$, if there exists an $n\in \mathbb {N}$, $M,\omega >0$ and a strongly continuous family of operator $(S(t))_{t\ge 0}$ in $L(\mathcal {X})$ with $\left\| S(t)\right\| \le M\textrm{e}^{\omega t}$ for all $t\ge 0$, $(\omega ,\infty )\in \rho (A)$ and

$$\begin{aligned} (\lambda I_\mathcal {X} -A)^{-1} x= \lambda ^{n-1} \int _0^\infty \textrm{e}^{-\lambda t}S(t)x\,\textrm{d}t \end{aligned}$$

for $$x\in \mathcal {X}$$.

It should be clear that for $n=1$ this coincides with the more known notion of a $C_0$-semigroup. The importance of integrated semigroups becomes clear when one is interested in the well-posedness of the Cauchy problem

26 $$\begin{aligned} {\left\{ \begin{array}{ll} \frac{d}{dt}x(t)=Ax(t),\quad t\ge 0,\\ x(0)=x_0, \end{array}\right. } \end{aligned}$$

for an $x_0\in \mathcal {X}$. Because then for all $x_0\in \mathcal {D}(A^n)$ the unique solution of (26) is given by

$$\begin{aligned} x(t)=S(t)A^{n-1}x_0 + \sum _{k=0}^{n-2} \frac{1}{k!} t^k A^k x_0 \end{aligned}$$

[15, eq. (4.1)]. Furthermore, for all $x_0 \in \mathcal {X}$ the function $t\mapsto S(t)x$ is a solution of the *n*-times integrated Cauchy problem and, thus, a *mild* solution of (26).

With this information we can illustrate the relation between the complex resolvent of a DAE and the generator of an integrated semigroup, given that (*E*, *A*) has a Weierstraß form.

### Theorem 3.9

Assume that (*E*, *A*) has a Weierstraß form as in (15). If $A_1$ is densely defined and (*E*, *A*) has a complex resolvent index $p_{\textrm{c, res}}^{(E,A)}$, then $A_1$ generates an (at least) $p_{\textrm{c, res}}^{(E,A)}+2$-times integrated semigroup.

### Proof

Assume that $$(E,A)\sim \left( \Big [{\begin{matrix} I_{\mathcal {Y}^1} &  0\\ 0 &  N \end{matrix}}\Big ], \Big [{\begin{matrix} A_1 &  0\\ 0 &  I_{\mathcal {Y}^2} \end{matrix}}\Big ]\right) $$ has a complex resolvent index $p=p_{\textrm{c,res}}^{(E,A)}$. Then, by (23) $\rho (E,A)=\rho (A_1)$ and

27 $$\begin{aligned} \Vert (\lambda I_{\mathcal {Y}^1}-A_1)^{-1} \Vert \le {\tilde{C}} \Vert (\lambda E-A)^{-1} \Vert \le C \Vert \lambda \Vert ^{p-1}, \quad \lambda \ge \omega , \end{aligned}$$

for given $C, {\tilde{C}}>0$ and $\omega >0$. Thus, $(I_{\mathcal {Y}^1},A_1)$ has a complex resolvent index and by [15, Cor. 4.9] $A_1$ generates an (at least) $p+2$-times integrated semigroup. $\square$

### Corollary 3.10

Assume that (*E*, *A*) has a complex radiality index. Then $A_1E_1^{-1}$ and $E_1^{-1} A_1$ from (20) and (21) generate integrated semigroups.

### Proof

In [2, Prop. 5.4] it was shown that the existence of the radiality index already implies the existence of the resolvent index. This implication extends naturally to the complex resolvent and complex radiality index. Therefore, if (*E*, *A*) has a complex radiality index, then by Theorem 3.3$$(E,A)\sim \left( \Big [{\begin{matrix} I_{\mathcal {Z}^1} &  0\\ 0 &  E_2A_2^{-1} \end{matrix}}\Big ], \Big [ {\begin{matrix} A_1E_1^{-1} &  0\\ 0 &  I_{\mathcal {Z}^2} \end{matrix}}\Big ]\right) \sim \left( \Big [{\begin{matrix} I_{\mathcal {X}^1} &  0\\ 0 &  A_2^{-1} E_2 \end{matrix}}\Big ], \Big [ {\begin{matrix} E_1^{-1} A_1 &  0\\ 0 &  I_{\mathcal {X}^2} \end{matrix}}\Big ]\right) $$ and by Theorem 3.9$A_1E_1^{-1}$ and $E_1^{-1} A_1$ generate integrated semigroups. $\square$

## Infinite-dimensional port-Hamiltonian DAEs

Let $\mathcal {X}, \mathcal {Z}$ be Hilbert spaces, $E, Q\in L(\mathcal {X},\mathcal {Z})$ and $A:\mathcal {D}(A)\subseteq \mathcal {Z}\rightarrow \mathcal {Z}$ be a closed and densely defined operator. Then, the following differential-algebraic equation

28 $$\begin{aligned} {\left\{ \begin{array}{ll} \frac{d}{d t} Ex(t) =AQx(t), \quad t\ge 0,\\ Ex(0) =z_0, \end{array}\right. } \end{aligned}$$

for a $z_0\in \mathcal {Z}$ is called a *port-Hamiltonian differential-algebraic equation* (short pH-DAE), if *A* is dissipative, $\textrm{ran}\, E$ is closed, *Q* is invertible and

29 $$\begin{aligned} E^*Q = Q^*E\ge 0 \end{aligned}$$

holds. In this context, we call

30 $$\begin{aligned} H(x):=\langle Ex,Qx\rangle _\mathcal {Z},\quad x\in \mathcal {X} \end{aligned}$$

the *Hamiltonian* of (*E*, *AQ*). Note that here $T:\mathcal {X}\rightarrow \mathcal {X}$ is called *non-negative*, denoted by $T\ge 0$, if $\langle Tx,x\rangle _\mathcal {X}\ge 0$ for all $x\in \mathcal {X}$.

These systems provide a framework for modelling and analysing energy-dissipating physical systems, including electrical circuits, mechanical systems, fluid dynamic, thermal systems or robotics and control systems. In this section we want to examine a few properties of pH-DAEs and show that the derivative of the Hamiltonian dissipates on all classical solutions of (28).

### Remark 4.1

- One can define a pH-DAE similarly for the case
  31 $$\begin{aligned} {\left\{ \begin{array}{ll} E\frac{d}{dt}x(t) =AQx(t), \quad t\ge 0,\\ x(0) =x_0, \end{array}\right. } \end{aligned}$$
  for $x_0\in \mathcal {X}$. Hence, *x* a *classical solution* of (31), if *x*(*t*) is continuously differentiable on $\mathbb {R}_{\ge 0}$, $x(t)\in \mathcal {D}(AQ)$ for all $t\ge 0$ and (31) holds.
- It can be observed that (29) implies
  32 $$\begin{aligned} EQ^{-1} = Q^{-*} E^*\ge 0. \end{aligned}$$
  In fact, using (29) we derive $EQ^{-1} = Q^{-*} Q^*EQ^{-1} = Q^{-*} E^*$, with $Q^{-*} = (Q^{-1})^*= (Q^*)^{-1}$. Applying the positivity of $EQ^*$ we have
  $$\begin{aligned} \langle Q^{-*} E^*z, z\rangle _\mathcal {Z} \!=\! \langle Q^{-*} E^*Q Q^{-1} z, z\rangle _\mathcal {Z} \!=\! \langle E^*Q (Q^{-1} z), (Q^{-1} z)\rangle _\mathcal {X} \!\ge \! 0, \quad z\!\in \!\mathcal {Z}. \end{aligned}$$
- Since *Q* is invertible it is possible to assume $\mathcal {Z}=\mathcal {X}$ and $Q=I_\mathcal {Z}$, such that *E* becomes non-negative and self-adjoint. This results from defining $z(t):=Qx(t)$ in (28), such that $\frac{d}{dt}Ex(t)=AQx(t)$ is equivalent to $\frac{d}{dt} EQ^{-1} z(t)=Az(t)$ and $EQ^{-1}$ being non-negative and self-adjoint as seen in (32). Similarly, $E\frac{d}{dt}x(t)=AQx(t)$ is equivalent to $E\frac{d}{dt}Q^{-1} z(t)=Az(t)$.
- By c) (28) is equivalent to $\frac{d}{dt}EQ^{-1} z(t) = Az(t)$. Since $\textrm{ran}\, E$ is closed, the same holds for $\textrm{ran}\, EQ^{-1}$ and one can split up the space into $\mathcal {Z}=\textrm{ran}\, EQ^{-1} \oplus \textrm{ker}\, EQ^{-1} =:\mathcal {Z}^1\oplus \mathcal {Z}^2$. Thus, $\frac{d}{dt}EQ^{-1} z(t) = Az(t)$ is equivalent to
  $$\begin{aligned} \frac{d}{dt}\begin{bmatrix} E_1 &  0\\ 0 &  0\end{bmatrix} \begin{bmatrix} z_1(t)\\ z_2(t)\end{bmatrix} = \begin{bmatrix} A_1 \\ A_2\end{bmatrix} \begin{bmatrix} z_1(t)\\ z_2(t)\end{bmatrix}, \quad t\ge 0, \end{aligned}$$
  whereby $A_i:\mathcal {Z} \rightarrow \mathcal {Z}^i$ and $E_1:=EQ^{-1}_{\textrm{ran}\, EQ^{-1}}$. Thus, the representation for port-Hamiltonian differential algebraic equations chosen here corresponds with the notion of an abstract Hamiltonian differential-algebraic equation defined in [13, Assumption 7]. Furthermore, it should be mentioned that there are more ways to define and approach port-Hamiltonian systems, such as using relations [4] or Dirac structures [24].

Next, we want to show that the derivative of the Hamiltonian dissipates on solutions, i.e. for a solution of (28), $\frac{d}{dt}H(x(t))\le 0$ for all $t\ge 0$.

### Proposition 4.2

Let $B\in L(\mathcal {Z})$ be a non-negative and self-adjoint operator on $\mathcal {Z}$ and assume that $\textrm{ran}\, B$ is closed. Then there exists a $c>0$ and $T\in L(\mathcal {Z})$ with $T\ge cI_\mathcal {Z}$ and

$$\begin{aligned} BTB=B. \end{aligned}$$

Furthermore, $\langle \cdot , \cdot \rangle _T:=\langle T \cdot , \cdot \rangle$ induces a norm on $\mathcal {Z}$ equivalent to $\Vert \cdot \Vert _\mathcal {Z}$.

Obviously, if *B* is invertible, then $T=B^{-1}$.

### Proof

Since $\textrm{ran}\, B$ is closed and *B* is self-adjoint, $\mathcal {Z}$ has an orthogonal decomposition into $\mathcal {Z}=\textrm{ran}\, B\,\oplus \,\textrm{ker}\, B$. Let $P_{\textrm{ran}\, B}$ and $P_{\textrm{ker}\, B}$ be the projections of $\mathcal {Z}$ onto $\textrm{ran}\, B$ and $\textrm{ker}\, B$. Define ${\tilde{B}} :\textrm{ran}\, B \rightarrow \textrm{ran}\, B$, ${\tilde{B}}(z) = Bz$. This is a bounded, positive self-adjoint operator on the Hilbert space $\textrm{ran}\, B$, which is bijective (note that $\textrm{ker}\, {\tilde{B}} = \textrm{ker}\, B \cap \textrm{ran}\, B = \{0\}$). Hence, since ${\tilde{B}}^{-1}$ is bounded, ${\tilde{B}}$ is strictly positive with

$$\begin{aligned} \langle {\tilde{B}} z_1, z_1 \rangle \ge c \Vert z_1\Vert ^2, \quad z_1\in \textrm{ran}\, B \end{aligned}$$

for all $z_1 \in \textrm{ran}\, B$, for some $c>0$.

Let $\iota _{\textrm{ran}\, B}:\textrm{ran}\, B\rightarrow \mathcal {Z}$ be the embedding and define

$$\begin{aligned} T:\iota _{\textrm{ran}\, B}{\tilde{B}}^{-1} \iota _{\textrm{ran}\, B}^*+ P_{\textrm{ker}\, B}. \end{aligned}$$

This operator satisfies $BTB=B$ and is bounded. Let $z= z_1+ z_2 \in \mathcal {Z}=\textrm{ran}\, B \, \oplus \textrm{ker}\, B$. Then

$$\begin{aligned} \langle Tz, z \rangle&= \langle {\tilde{B}} z_1 + z_2 , z_1 + z_2 \rangle \\&= \langle {\tilde{B}} z_1 , z_1 \rangle + \langle z_2 ,z_2 \rangle \\&\ge c \Vert z_1\Vert ^2 + \Vert z_2\Vert ^2 . \end{aligned}$$

Thus, *T* is strictly positive. The self-adjointness of *T* follows from that of *B*, completing the proof. $\square$

### Corollary 4.3

Let $E,Q\in L(\mathcal {X},\mathcal {Z})$, whereby *E* has a closed range, *Q* is invertible and (29) holds. Then, there exists a $c>0$ and $T\in L(\mathcal {Z})$ with $T\ge cI_\mathcal {Z}$ and

33 $$\begin{aligned} E^*TE = E^*Q = Q^*E. \end{aligned}$$

Furthermore, $\langle \cdot , \cdot \rangle _T :=\langle T\cdot , \cdot \rangle$ induces a norm on $\mathcal {Z}$ equivalent to $\Vert \cdot \Vert _\mathcal {Z}$.

### Proof

This follows from Remark 4.1, Proposition 4.2 with $B=EQ^{-1}$ and the fact that *Q* is invertible. $\square$

### Theorem 4.4

Let (*E*, *AQ*) be a pH-DAE with Hamiltonian *H*. Then, for all classical solutions $x:\mathbb {R}_{\ge 0}\rightarrow \mathcal {X}$

$$\begin{aligned} \frac{d}{dt}\langle E x(t), Qx(t)\rangle \le 0, \quad t\ge 0. \end{aligned}$$

### Proof

By Corollary 4.3 we know that $T\ge cI_Z$, such that (33) holds. Let $x:\mathbb {R}_{\ge 0}\rightarrow \mathcal {X}$ be a classical solution of (28). Then

$$\begin{aligned} \frac{d}{dt}\langle Ex(t), Qx(t) \rangle _\mathcal {Z}&= \frac{d}{dt} \langle x(t), \underbrace{E^*Q}_{=E^*T E} x(t)\rangle _\mathcal {Z} \\&= 2\textrm{Re} \left\langle Ex(t), \frac{d}{dt} TEx(t)\right\rangle _\mathcal {Z} \\&= 2\textrm{Re} \left\langle Qx(t), \frac{d}{dt}Ex(t)\right\rangle _\mathcal {Z}\\&= 2\textrm{Re} \left\langle Qx(t), AQx(t)\right\rangle _\mathcal {Z} \le 0, \quad t\ge 0, \end{aligned}$$

where we used the continuity of *E*, *Q* and *T* in the second and third equation. Note that we used $\langle Ex(t) = TEx(t)\rangle = \langle x(t), E^*T E x(t) \rangle = \langle x(t), Q^*Ex(t) \rangle = \langle Qx(t), Ex(t)\rangle$ in the third equality. $\square$

### Remark 4.5

From the proof of Theorem 4.4 it becomes clear that for all classical solutions *x* of (28) the DAE is already dissipating, i.e. one has

$$\begin{aligned} \textrm{Re} \langle E x(t), AQx(t)\rangle _T \le 0, \quad t\ge 0. \end{aligned}$$

It is possible to show the same for the adjoint system and, if the set containing all trajectories of the solutions of (28) is closed, one can apply [7, Thm. 3.7] to gain a Weierstraß form on a subset. To be more precise, we know that $\textrm{ran}\, E^*$ is closed and $Q^{-1}$ is invertible. Thus, there also exists a $S\in L(\mathcal {X})$ with $S\ge {\tilde{c}} I_\mathcal {X}$, for a ${\tilde{c}}>0$, with

$$\begin{aligned} ESE^*= EQ^{-1} = Q^{-*} E^*\end{aligned}$$

and $\langle \cdot ,\cdot \rangle _{S^{-1}} = \langle S^{-1} \cdot , \cdot \rangle$ induces a norm on $\mathcal {X}$ equivalent to $\Vert \cdot \Vert _\mathcal {X}$. Define $\mathcal {X}_{S^{-1}}:=(\mathcal {X}, \langle \cdot ,\cdot \rangle _{S^{-1}})$ and $\mathcal {Z}_T:=(\mathcal {Z}, \langle \cdot ,\cdot \rangle _{T})$. Let $z\in \{z\in \mathcal {Z} \,\, \vert \, Tz\in \mathcal {D}(A^*)\}$ and $x\in \mathcal {D}(AQ)$. Then,

$$\begin{aligned} \langle AQx,z\rangle _T = \langle x, SQ^*A^*Tz\rangle _{S^{-1}},. \end{aligned}$$

withIn fact, by simple reformulations it is easy to see that

$$\begin{aligned} \mathcal {D}((AQ)^{*}_{S^{-1}, T})=\{z\in \mathcal {Z} \,\, \vert \,\, \exists x^*\in \mathcal {X}, \forall x \in \mathcal {D}(AQ):\langle z,AQx\rangle _T=\langle x^*,x\rangle _{S^{-1}} \}, \end{aligned}$$

where $(AQ)^{*}_{S^{-1}, T}$ denotes the adjoint of $AQ:\mathcal {D}(AQ)\subseteq \mathcal {X}_{S^{-1}}\rightarrow \mathcal {Z}_T$. Consider now the *adjoint system* of (28)

34 $$\begin{aligned} {\left\{ \begin{array}{ll} \frac{d}{dt} E^*z(t)= Q^*A^*z(t), \quad t\ge 0,\\ E^*z(0) = x_0\in \mathcal {X}. \end{array}\right. } \end{aligned}$$

Then, by choosing $z(t)=T{\tilde{z}}(t)$, this is equivalent to

35 $$\begin{aligned} {\left\{ \begin{array}{ll} \frac{d}{dt} (E)^{*}_{S^{-1}, T} {\tilde{z}}(t) = (AQ)^{*}_{S^{-1}, T} {\tilde{z}}(t), \quad t\ge 0,\\ (E)^{*}_{S^{-1}, T} {\tilde{z}}(0) = {\tilde{x}}_0\in \mathcal {X}. \end{array}\right. } \end{aligned}$$

Let *z* be a classical solution of (35). Assuming that $A^*$ is dissipative, one has

$$\begin{aligned} \textrm{Re}\langle (E)^{*}_{S^{-1}, T} z(t), (AQ)^{*}_{S^{-1}, T} z(T)\rangle _{S ^{-1}}&= \textrm{Re}\langle E^*T z(t), SQ^*A^*Tz(T)\rangle _\mathcal {X} \\&= \textrm{Re}\langle E^*T z(t), \frac{d}{dt} S E^*T z(T)\rangle _\mathcal {X}\\&= \textrm{Re}\langle T z(t), \frac{d}{dt} E S E^*T z(T)\rangle _\mathcal {X}\\&= \textrm{Re}\langle Q^{-1} T z(t), S^{-1} \frac{d}{dt} S E^*T z(T)\rangle _\mathcal {X}\\&= \textrm{Re}\langle Q^{-1} T z(t), S^{-1} S Q^*A^*T z(T)\rangle _\mathcal {X}\\&= \textrm{Re}\langle T z(t), A^*T z(T)\rangle _\mathcal {X} \le 0, \quad t\ge 0. \end{aligned}$$

Thus, if $U:=\{u\in \mathcal {Z}\,\vert \, \exists x$ classical solution of (28) $\exists t\ge 0:x(t)=u \}\subseteq \mathcal {Z}$ and $V :=\{v\in \mathcal {Z}\,\vert \, \exists z$ classical solution of (35) $\exists t\ge 0:z(t)=v \}\subseteq \mathcal {Z}$ are both closed sets, one can apply [7, Thm. 3.7] to $(E\vert _U, (AQ)\vert _U)$ on the spaces $\mathcal {X}_{S^{-1}}$ and $\mathcal {Z}_T$.

### Example 4.6

Consider longitudinal vibrations in a viscoelastic nanorod. Let *l* be the length of the nanorod, *N*(*x*, *t*) be the resultant force of axial stress, *w*(*x*, *t*) be the displacement of the nanorod in *x* direction, *C* be the elastic modulus, *D* be the cross sectional area, $\mu$ be a non-local parameter, $\rho$ the mass density, $\tau _d$ the viscous damping and $a^2$, $b^2$ be the stiffness and damping coefficients of the light viscoelastic layer and consider the system introduced in [9]. Consider

36a $$\begin{aligned} \frac{\partial N(x,t)}{\partial x}&= a^2 w(x,t) + b^2 \frac{\partial w(x,t)}{\partial t} + \rho D \frac{\partial ^2 w(x,t)}{\partial t^2}, \end{aligned}$$

36b $$\begin{aligned} N(x,t)-\mu \frac{\partial ^2 N(x,t)}{\partial x^2}&= CD\left( \frac{\partial w(x,t)}{\partial x} + \tau _d \frac{\partial ^2 w(x,t)}{\partial x\partial t}\right) , \end{aligned}$$

with boundary conditions

37 $$\begin{aligned} \frac{\partial w}{\partial t}(0,t) = \frac{\partial w}{\partial t}(l,t)=0. \end{aligned}$$

In [6] the associated port-Hamiltonian system is given through38and state

39 $$\begin{aligned} z(x,t)=\begin{bmatrix} w(x,t)\\ \rho D \frac{\partial w(x,t)}{\partial t}\\ \mu \rho D \frac{\partial ^2w(x,t)}{\partial x\partial t}\\ \frac{\partial w(x,t)}{\partial x}\\ N(x,t) \end{bmatrix}. \end{aligned}$$

Here, *E* and *Q* are bounded operators living on $\mathcal {X}=\mathcal {Z}=L^2((0,l); \mathbb {R}^5)$ and $A:\mathcal {D}(A)\subseteq \mathcal {X}\rightarrow \mathcal {X}$ with

$$\begin{aligned} \mathcal {D}(A):=\{ (z_1,\ldots ,z_5)^T \in \mathcal {X} \, \vert \, z_2, z_5 \in H^1(0,l), z_2(0)=z_2(l)=0 \}. \end{aligned}$$

In [6] the existence of solution was studied by reducing the system to a homogeneous port-Hamiltonian system (see [8, Ch. 7]). Here, we provide another approach by simply examining the dissipativity of the DAE as seen in Remark 4.5.

Since the various physical constants in *Q* are positive, it is easy to show that $E^*Q$ is non-negative and self-adjoint and with

$$\begin{aligned} \langle Az,z\rangle _\mathcal {X}&= \int _0^l z_2(x)\frac{\partial z_5(x)}{\partial x} + z_5(x)\frac{\partial z_2(x)}{\partial x} - b^2 z_2^2(x) - (CD\tau _d + \mu b^2)z^2_3(x)\,\text {d}x\\  &= -b^2 \left\| z_2\right\| _\mathcal {X}^2 - (CD\tau _d + \mu b^2)\left\| z_3\right\| _\mathcal {X}^2 \le 0, \quad z=(z_1,\ldots ,z_5)\in \mathcal {D}(A), \end{aligned}$$

*A* is dissipative. Thus, (*E*, *AQ*) fits into our definition of a port-Hamiltonian DAE.

Now, as in [6, p. 452] we impose the boundary conditions directly on the space and show that (38) has radiality index 0. Define

$$\begin{aligned}  &   \mathcal {X}_t:=\{ (z_1,\ldots ,z_5)^T \in L^2((0,l);\mathbb {R}^5) \, \vert \, z_2, z_5 \in H^1(0,l), z_2(0)\\  &   \quad =z_2(l)=0, \mu \frac{\partial z_2(x)}{\partial x} = z_3(x) \}. \end{aligned}$$

with the inner product $\langle \cdot , \cdot \rangle _{\tilde{\mathcal {X}}_t}:=\langle Q\cdot ,\cdot \rangle _{\mathcal {X}_t}$. Since *Q* is coercive this induces a norm equivalent to $\Vert \cdot \Vert _{\mathcal {X}}$. $\mathcal {X}_t$ is in fact closed [6, Lem. 4.1]. Define $\tilde{\mathcal {X}} :=(\mathcal {X}, \langle \cdot , \cdot \rangle _{\tilde{\mathcal {X}}_t})$. Then,

40 $$\begin{aligned} \text {Re} \langle AQz, Ez\rangle _{\tilde{\mathcal {X}}_t}&\le \text {Re} \int _0^l \frac{1}{\rho D} \left( z_2(x)\frac{\partial z_5(x)}{\partial x} + z_5(x)\frac{\partial z_2(x)}{\partial x} \right) \,\text {d}x=0 \end{aligned}$$

holds for all $z=(z_1,\ldots ,z_5)\in \mathcal {D}(AQ)$. Furthermore, $(AQ)^*= A^*Q$ in $\mathcal {X}_t$ and, through similar calculations, one derives $\textrm{Re}\langle A^*Q z, Ez\rangle _{\mathcal {X}_t}\le 0$ for all $z\in \mathcal {D}((AQ)^*)$. Then, by [7, Thm. 3.6, 3.7] (*E*, *AQ*) is 0-radial and admits a decomposition as in (20) on $\mathcal {X}_t = \mathcal {X}_t^1\oplus \mathcal {X}_t^2$ with $E_2(AQ)_2^{-1} = 0$ and $(AQ)_2^{-1} E_2=0$. In particular, $(AQ)_1E_1^{-1}$ generates a contraction semigroup on $\mathcal {X}_t^1$.

Note that in this example the operator *T* from above is already given by *Q*, since $E^*Q E = E^*Q = Q^*E$.

By [7, Thm. 3.7] it becomes clear that if (*E*, *AQ*) is a pH-DAE with $\textrm{Re} \langle AQx, Ex\rangle \le 0$ and $\textrm{Re} \langle (AQ)^*z, E^*z\rangle \le 0$, then (*E*, *AQ*) is in fact 0-radial (and therefore has radiality index 0), admits a Weierstraß form as in (20) or (21) and $(AQ)_1E_1^{-1}$ and $E_1^{-1} (AQ)_1$ generate contraction semigroups on $\tilde{\mathcal {Z}}^1$ and $\tilde{\mathcal {X}}^1$. In this case, $(AQ)_1E_1^{-1}$ and $E_1^{-1} (AQ)_1$ also generate $C_0$-semigroups on $\mathcal {Z}^1$ and $\mathcal {X}^1$.

## Weierstraß form and solutions of port-Hamiltonian DAEs

It is possible to show that the (complex) resolvent index for pH-DAEs always exists and that it is bounded by 2 (3). This is an already widely known result in finite dimensions and was shown for the infinite-dimensional case in [2] for $\mathcal {X}=\mathcal {Z}$ and $Q=I$.

### Proposition 5.1

Let *E*, *Q* and *A* be defined as before, such that (*E*, *AQ*) defines a pH-DAE. Assume that there exists a $\omega >0$ with $(\omega ,\infty )\subseteq \rho (E,AQ)$ $(\mathbb {C}_{\textrm{Re} >\omega }\subseteq \rho (E,AQ))$. Then the (complex) resolvent index exists and is at most 2 (at most 3).

### Proof

Using Remark 4.1 and [2, Thm. 3.3] one knows that $(EQ^{-1}, A)$ is a pH-DAE with an existing (complex) resolvent index, which is at most 2 (at most 3), and together with $(\lambda EQ^{-1} -A)^{-1}=Q(\lambda E-AQ)^{-1}$ for all $\lambda \in \rho (EQ^{-1}, A) = \rho (E,AQ)$ the assertion follows. $\square$

### Proposition 5.2

Let *E*, *Q* and *A* be defined as before, such that (*E*, *AQ*) defines a pH-DAE and assume that there exists a $\omega >0$ with $\mathbb {C}_{\textrm{Re} >\omega }\subseteq \rho (E,AQ)$. Then, for every $x_0 \in \textrm{ran}\,((\mu E-AQ)^{-1} E)^{p_{\textrm{c,res}}^{(E,AQ)}+2}$ there exists a unique solution $x:\mathbb {R}_{\ge 0}\rightarrow \mathcal {X}$ of the following differential algebraic equation

$$\begin{aligned} {\left\{ \begin{array}{ll} \frac{d}{dt}Ex(t) = AQx(t), \quad t\ge 0,\\ x(0) =x_0. \end{array}\right. } \end{aligned}$$

### Proof

This is a direct consequence of Theorem 2.2 and Proposition 5.1. $\square$

The next goal is to examine the well-posedness of a pH-DAE on the whole domain, given that the radiality index exists. Let (*E*, *AQ*) be such a pH-DAE. By Theorem 3.3 (28) is equivalent to

41 $$\begin{aligned} \frac{d}{dt}\begin{bmatrix} I_{\mathcal {Z}^1}^{-1} &  0\\ 0 &  E_2(AQ)_2^{-1} \end{bmatrix} \begin{bmatrix} z_1(t) \\ z_2(t) \end{bmatrix} = \begin{bmatrix} (AQ)_1E_1^{-1} &  0 \\ 0 &  I_{\mathcal {Z}^2} \end{bmatrix} \begin{bmatrix} z_1(t) \\ z_2(t) \end{bmatrix}, \quad t\ge 0 \end{aligned}$$

and (31) is equivalent to

42 $$\begin{aligned} \begin{bmatrix} I_{\mathcal {X}^1}^{-1} &  0\\ 0 &  (AQ)_2^{-1} E_2 \end{bmatrix} \frac{d}{dt} \begin{bmatrix} x_1(t) \\ x_2(t) \end{bmatrix} = \begin{bmatrix} E_1^{-1}(AQ)_1 &  0 \\ 0 &  I_{\mathcal {X}^2} \end{bmatrix} \begin{bmatrix} x_1(t) \\ x_2(t) \end{bmatrix}, \quad t\ge 0. \end{aligned}$$

The main problem with a transformation like that is that (41) and (42) are not necessarily pH-DAEs anymore. Thus, the resulting question is under which circumstances $(AQ)_1E_1^{-1}$ and $E_1^{-1}(AQ)_1$ generate a $C_0$-semigroup. A possible condition for that is *AQ* being strongly (*E*, *p*)-radial [19, Thm. 2.6.1].

### Theorem 5.3

Let *E*, *Q* and *A* be defined as before, such that (*E*, *AQ*) defines a pH-DAE. Assume that the radiality index exists and that $\mathbb {C}_{\textrm{Re} \ge \omega }\subseteq \rho (E,AQ)$ for a $\omega >0$. Then, $(AQ)_1E_1^{-1}$ and $E_1^{-1}(AQ)_1$ generate an (at least) $p_{\textrm{c,res}}^{(E,AQ)}+2$-times integrated semigroup. If additionally

- $Q^*(\mathcal {Z}^1) = \mathcal {X}^1$, then $E_1^{-1} (AQ)_1$ generates a $C_0$-semigroup on $\mathcal {X}^1$.
- $Q(\mathcal {X}^1)=\mathcal {Z}^1$, then $(AQ)_1 E_1^{-1}$ generates a $C_0$-semigroup on $\mathcal {Z}^1$.

### Proof

Let $x\in \mathcal {X}$ and $\lambda \in \rho (E,AQ)$ with $\lambda >0$. Thus, $(\lambda E-AQ)^{-1} \in L(\mathcal {Z}, \mathcal {X})$. As seen in the proof of Theorem 3.3$(AQ)_1E_1^{-1}$ and $E_1^{-1} (AQ)_1$ are densely defined and by Proposition 5.1 the complex resolvent index $p_{\textrm{c,res}}^{(E,AQ)}$ exists. Thus, by Theorem 3.9$(I_{\mathcal {Z}^1}, (AQ)_1E_1^{-1})$ and $(I_{\mathcal {X}^1}, E_1^{-1} (AQ)_1)$ generate an (at least) $p_{\textrm{c, res}}^{(E,AQ)}+2$-times integrated semigroup.

In order to show a), assume that $Q^*(\mathcal {Z}^1) = Q^*(E(\mathcal {X}^1))= \mathcal {X}^1$ holds. Thus, $Q^*E:\mathcal {X}^1\rightarrow \mathcal {X}^1$ is non-negative and self-adjoint. In fact, since $Q^*E$ is self-adjoint the mappings $Q^*E \pm {\textrm{i}} I_\mathcal {X}$ are surjective. Hence, for every $z\in \mathcal {X}^1$ there are $x_\pm = x_\pm ^1+x_\pm ^2 \in \mathcal {X}=\mathcal {X}^1\oplus \mathcal {X}^2$ with $(Q^*E\pm {\textrm{i}} I_\mathcal {X})x_\pm = z$. Together with $Q^*E(\mathcal {X}^1)=\mathcal {X}^1$ one has $PQ^*E x_\pm = PQ^*E x_\pm ^1 + PQ^*Ex_\pm ^2 = Q^*E Px_\pm ^1 = Q^*E Px_\pm$ and

$$\begin{aligned} z&= Pz\\&= PQ^*E x_\pm \pm {\textrm{i}} Px_\pm \\&= Q^*E Px_\pm \pm {\textrm{i}} Px_\pm = (Q^*E \pm {\textrm{i}} I_{\mathcal {X}^1}) x_\pm . \end{aligned}$$

Thus, $Q^*E \pm {\textrm{i}} I_{\mathcal {X}^1}:\mathcal {X}^1\rightarrow \mathcal {X}^1$ are surjective and consequently, as a closed operator, $(Q^*E)\vert _{\mathcal {X}^1}$ is self-adjoint. Furthermore, since $Q^*$ and $E_1$ are invertible and $(Q^*E)\vert _{\mathcal {X}^1}$ is self-adjoint, $(Q^*E)\vert _{\mathcal {X}^1}$ becomes invertible and $((Q^*E)\vert _{\mathcal {X}^1})^{-1}$ self-adjoint as well. Hence, using Lemma 4.2 for $B=((Q^*E)\vert _{\mathcal {X}^1})^{-1}$ there exists a $c>0$, such that $T=((Q^*E)\vert _{\mathcal {X}^1})\ge cI_{\mathcal {X}^1}$ and $\langle \cdot , \cdot \rangle _{\tilde{\mathcal {X}}^1}:=\langle (Q^*E)\vert _{\mathcal {X}^1} \cdot ,\cdot \rangle$ induces a norm equivalent to $\Vert \cdot \Vert _\mathcal {X}$ on $\mathcal {X}^1$. Define $\tilde{\mathcal {X}}^1:=(\mathcal {X}^1, \langle \cdot , \cdot \rangle _{\tilde{\mathcal {X}}^1})$. Since *A* is dissipative one derives

$$\begin{aligned} \textrm{Re} \langle E_1^{-1}(AQ)_1 x, x\rangle _{\tilde{\mathcal {X}}^1} = \textrm{Re} \langle AQx, Qx\rangle _\mathcal {X} \le 0, \quad x\in \mathcal {D}(E_1^{-1} (AQ)_1), \end{aligned}$$

which means that $E_1^{-1}(AQ)_1$ is dissipative in $\tilde{\mathcal {X}}^1$. Given that $\mathcal {X}=\mathcal {X}^1\oplus \mathcal {X}^2$, $E_1$ is bijective and $(\lambda E_1 -(AQ)_1)$ is surjective, $(\lambda I_{\mathcal {X}^1}-E_1^{-1}(AQ)_1) = E_1^{-1}(\lambda E_1-(AQ)_1):\mathcal {D}((AQ)_1)\subseteq \mathcal {X}^1\rightarrow \mathcal {X}^1$ becomes surjective as well.

By the theorem of Lumer-Phillips [1, Thm.3.4.5] $E_1^{-1}(AQ)_1$ generates a contraction semigroup on $\tilde{\mathcal {X}}^1$. In this case, $E_1^{-1}(AQ)_1$ also generates a $C_0$-semigroup on $\mathcal {X}^1$.

To show b) assume that $Q(\mathcal {X}^1)=Q(E^{-1} (\mathcal {Z}^1))=\mathcal {Z}^1$ holds. As seen before it is possible to show that $QE^{-1}$ is non-negative, self-adjoint and has a non-negative and self-adjoint inverse, such that $QE^{-1}\ge cI_{\mathcal {X}}$ for a $c>0$. Define $\langle \cdot ,\cdot \rangle _{\tilde{\mathcal {Z}}^1}:=\langle QE^{-1} \cdot , \cdot \rangle$ and $\tilde{\mathcal {Z}}^1 :=(\mathcal {Z}^1, \langle \cdot ,\cdot \rangle _{\tilde{\mathcal {Z}}^1})$. Thus,

$$\begin{aligned} \textrm{Re} \langle (AQ)_1E_1^{-1} z, z \rangle _{\tilde{\mathcal {Z}}^1} = \textrm{Re}\langle A QE_1 z, QE_1 z\rangle \le 0,\quad z\in \mathcal {D}((AQ)_1E_1^{-1}) \end{aligned}$$

since *A* is dissipative. Then, the rest of this proof follows the previous part. $\square$

### Remark 5.4

Let $\mathcal {X}=\mathcal {Z}$ and $Q=I_\mathcal {X}$ and assume that *E* commutes with *A* on $\mathcal {D}(A)$. Thus

$$\begin{aligned} Ex=E(\lambda E-A)(\lambda E-A)^{-1} x = (\lambda E-A)E (\lambda E-A)^{-1} x \end{aligned}$$

or equivalently $(\lambda E-A)^{-1} Ex=E(\lambda E-A)^{-1} x$. Thus, the left- and right-*E* resolvent coincide and $\mathcal {X}^1=\mathcal {Z}^1$, $\mathcal {X}^2=\mathcal {Z}^2$ as well as the projections from Theorem 3.3$P=R$. In this case $Q^*E(\mathcal {X}^1)=\mathcal {X}^1$ and $QE^{-1}(\mathcal {Z}^1)=\mathcal {Z}^1$ hold. Unfortunately, the assumption that *E* and *AQ* commute massively limits the choice of systems. Because if one considers the following type of system

$$\begin{aligned} \frac{d}{dt} \underbrace{\begin{bmatrix} I &  0\\ 0 &  N \end{bmatrix}}_{=:E} \begin{bmatrix} x_1(t) \\ x_2(t) \end{bmatrix} = \underbrace{\begin{bmatrix} A_1 &  A_2 \\ A_3 &  A_4 \end{bmatrix}}_{=:A} \begin{bmatrix} x_1(t) \\ x_2(t) \end{bmatrix}, \quad t\ge 0, \end{aligned}$$

and additionally assume that $EA=AE$ hold, then $A_2=A_2N$, $NA_3=A_3$ and $NA_4=A_4N$.

### Example 5.5

Consider the system

$$\begin{aligned} \frac{d}{dt}\underbrace{\begin{bmatrix} I &  0\\ 0 &  0 \end{bmatrix}}_{=\mathcal {E}} \begin{bmatrix} x_1(t)\\ x_2(t) \end{bmatrix} = \underbrace{\begin{bmatrix} A_1 &  A_2\\ A_3 &  A_4 \end{bmatrix}}_{=\mathcal {A}} \begin{bmatrix} x_1(t)\\ x_2(t) \end{bmatrix} \end{aligned}$$

with existing radiality index and $\mathcal {A}$ being dissipative.

Since $\textrm{ker}\, E\subseteq \mathcal {X}^2$ and $\mathcal {X}=\mathcal {X}^1\oplus \mathcal {X}^2$, one has $\mathcal {E}_1=I_{\mathcal {X}^1}$. Thus, one has $\mathcal {E}(\mathcal {X}^1)= \mathcal {X}^1 = \mathcal {Z}^1 = \mathcal {E}_1(\mathcal {Z}^1)$. But, in this case, it is even possible to say directly something about the generation of $C_0$-semigroups without using Theorem 5.3. Because in such a case $\mathcal {A}_1 = \mathcal {A}_1\mathcal {E}_1^{-1} = \mathcal {E}_1^{-1}\mathcal {A}_1$ is dissipative (as a restriction of a dissipative operator) and $\lambda I_{\mathcal {X}^1}-\mathcal {A}_1$ is surjective (since $(\omega ,\infty )\subseteq \rho (\mathcal {E},\mathcal {A})=\rho (I_{\mathcal {X}^1}, \mathcal {A}_1)$). Hence, using the Lumer-Phillips theorem one obtains the same outcome. This result is similar to [7, Sect. IV].

### Example 5.6

Recall the system from [2, Ex. 3.4]. Define $A=\mathrm{diag\,} (A_0, A_1, A_2,\ldots )$ with

$$\begin{aligned} A_0=\begin{bmatrix} 0 &  -1\\ 1&  0\end{bmatrix},\quad A_k=\begin{bmatrix} 0 &  \sqrt{k^4+1} \\ -\sqrt{k^4+1} &  -2\end{bmatrix}, \quad k\in \mathbb {N}, \end{aligned}$$

and $\mathcal {D}(A):=\{x\in \ell ^2 \, \vert \, Ax\in \ell ^2\}$. Then *A* can be extended to $\ell ^2$, which will denoted by $A_{-1}$. Define $E \in L(\ell ^2)$, $B:\mathbb {R}\rightarrow \mathcal {D}(A^*)'$ and $C:\mathcal {D}(A)\rightarrow \mathbb {R}$ with $E=\mathrm{diag\,}(E_0, E_1, E_2,\ldots )$ and $B=(B_0, B_1, B_2, \ldots )^T = C^*$, where

$$\begin{aligned} E_0&= \begin{bmatrix} 1 &  0\\ 0 &  0\end{bmatrix},&E_k&= \begin{bmatrix} 1 &  0 \\ 0 &  1\end{bmatrix}, \quad k\in \mathbb {N},&\\ B_0&= \begin{bmatrix} 0\\ 1\end{bmatrix},&B_k&=\begin{bmatrix} 0\\ k^{\frac{5}{4}}\end{bmatrix}, \quad k\in \mathbb {N}.&\end{aligned}$$

Define then the system

$$\begin{aligned} \frac{{\hbox {d}}}{{\hbox {d}}t}\underbrace{\begin{bmatrix} E &  0 &  0\\ 0 &  0 &  0\\ 0 &  0 &  0\end{bmatrix}}_{\mathcal {E}} \begin{bmatrix} x_1\\ x_2\\ x_3\end{bmatrix} = \underbrace{\begin{bmatrix} A_{-1} &  B &  0\\ -C &  0 &  I\\ 0 &  -I &  0\end{bmatrix}}_{\mathcal {A}} \begin{bmatrix} x_1\\ x_2 \\ x_3 \end{bmatrix} \end{aligned}$$

on $\mathcal {X}=\ell ^2\times \mathbb {R}\times \mathbb {R}$. Obviously, $\mathcal {E}$ is non-negative and self-adjoint and, by its construction, $\mathcal {A}$ with maximal domain is dissipative. Furthermore, for $\lambda \in \rho (\mathcal {E}, \mathcal {A})$

$$\begin{aligned} (\lambda \mathcal {E}-\mathcal {A})^{-1} = \begin{bmatrix} (\lambda E-A)^{-1} &  0 &  (\lambda E-A)^{-1} B \\ 0 &  0 &  I \\ -C(\lambda E-A)^{-1} &  -I &  C(\lambda E-A)^{-1} B\end{bmatrix}. \end{aligned}$$

It was shown in [2, Ex. 3.4] that this system has real resolvent index 2 and complex resolvent index 3.

We now compute the radiality index of $(\mathcal {E}, \mathcal {A})$. First, calculate

$$\begin{aligned} (\lambda \mathcal {E}-\mathcal {A})^{-1} \mathcal {E}&= \begin{bmatrix} (\lambda E-A)^{-1} E &  0 &  0 \\ 0 &  0 &  0 \\ -C(\lambda E-A)^{-1} E &  0 &  0\end{bmatrix}, \\ \mathcal {E} (\lambda \mathcal {E}-\mathcal {A})^{-1}&= \begin{bmatrix} E(\lambda E-A)^{-1} &  0 &  E(\lambda E-A)^{-1} B \\ 0 &  0 &  0 \\ 0 &  0 &  0 \end{bmatrix}. \end{aligned}$$

It is needed to determine the growth rate of $(\lambda E-A)^{-1} = \textrm{diag}(M_0(\lambda ), M_1(\lambda ),$ $M_2(\lambda ),\ldots )$ with

$$\begin{aligned}  &   M_0(\lambda ) = \begin{bmatrix} 0 &  -1\\ 1&  \lambda \end{bmatrix}, \quad \\  &   M_k(\lambda ) = \frac{1}{\lambda (\lambda +2)+k^4+1}\begin{bmatrix} \lambda +2 &  \sqrt{k^4+1} \\ -\sqrt{k^4+1} &  \lambda \end{bmatrix}, \quad k\in \mathbb {N}. \end{aligned}$$

It is easy to see that $M_0(\lambda )$ has a linear growth and $M_k(\lambda )$ is decreasing with rate $\frac{1}{\lambda }$. Hence, due to the structure of *E*, $(\lambda E-A)^{-1} E$ and $E(\lambda E-A)^{-1}$ are bounded and

$$\begin{aligned} \left\| (\lambda E-A)^{-1} E (\mu E-A)^{-1} E \right\| = \left\| E(\lambda E-A)^{-1} E (\mu E-A)^{-1} \right\| \le \frac{C}{\lambda \mu }, \quad \lambda , \mu >0 \end{aligned}$$

for a given $C>0$. Moreover, by simple calculations it is easy to show that $B_k^T M_k(\lambda )$ and $M_k(\lambda )B_k$ are still decreasing with rate $\frac{1}{\lambda }$. Thus, for all $\lambda , \mu >0$

$$\begin{aligned} \left\| C(\lambda E-A)^{-1} E (\mu E-A)^{-1} E \right\|&\le \frac{C}{\lambda \mu }, \\ \left\| E(\lambda E-A)^{-1} E (\mu E-A)^{-1} B \right\|&\le \frac{C}{\lambda \mu }. \end{aligned}$$

Since

$$\begin{aligned}&(\lambda \mathcal {E}-\mathcal {A})^{-1} \mathcal {E} (\mu \mathcal {E}-\mathcal {A})^{-1} \mathcal {E} = \begin{bmatrix} (\lambda E-A)^{-1} E(\mu E-A)^{-1} E &  0 &  0 \\ 0 &  0 &  0 \\ -C(\lambda E-A)^{-1} E(\mu E-A)^{-1} E &  0 &  0\end{bmatrix}, \\&\mathcal {E} (\lambda \mathcal {E}-\mathcal {A})^{-1} \mathcal {E} (\mu \mathcal {E}-\mathcal {A})^{-1} \\&\quad = \begin{bmatrix} E(\lambda E-A)^{-1} E(\mu E-A)^{-1} &  0 &  E(\lambda E-A)^{-1} E(\mu E-A)^{-1} B \\ 0 &  0 &  0 \\ 0 &  0 &  0 \end{bmatrix} \end{aligned}$$

the system $(\mathcal {E}, \mathcal {A})$ has radiality index 1.

## Data Availability

No data were used for the research described in the article.
